# Comparison of the Prevention of Esophageal Stricture Between Oral Prednisolone Alone and Oral Prednisolone Combined with Nasogastric Tube in Superficial Esophageal Cancer After Endoscopic Submucosal Dissection

**DOI:** 10.5152/tjg.2024.23487

**Published:** 2024-06-01

**Authors:** Lihong Teng, Xiaoyun Yang, Yiping Hong, Jin Ding

**Affiliations:** Department of Gastroenterology, Jinhua Hospital of Zhejiang University School of Medicine, Wucheng, Jinhua, China

**Keywords:** Early esophageal cancer, esophageal stricture, endoscopic submucosal dissection, steroid, nasogastric tube

## Abstract

**Background/Aims:**

There is a lack of effective and safe methods for preventing esophageal stricture after large endoscopic submucosal dissection (ESD) in patients with superficial esophageal cancer. We aimed to compare the effectiveness of oral prednisolone alone versus a combination of oral prednisolone and nasogastric tube in preventing esophageal stricture following extensive ESD.

**Materials and Methods:**

We retrospectively gathered clinical data from a single center on patients with early esophageal cancer who underwent ESD. Patients were categorized into 2 groups: the steroid group (receiving only oral prednisolone) and the steroid+nasogastric tube retention (NGT) group. We analyzed the incidence of esophageal stricture and identified risk factors for its development.

**Results:**

The study included 79 patients, with 30 in the steroid group and 49 in the steroid + NGT group. The incidence of stricture was significantly higher in the steroid group (9/30, 30.0%) compared to the steroid + NGT group (3/49, 6.1%) (*P* = .004). Notably, we observed a significant difference in the stricture rates between the 2 groups, particularly in patients with a complete circumferential defect (100% and 16.7%) (*P* = .015). Multivariate logistic regression analysis revealed that a full circumferential defect of the esophageal mucosa (OR 12.501; 95% CI 1.907, 81.047; *P* = .008), invasion depth beyond the lamina propria (OR 5.635; 95% CI 1.039, 30.559; *P* = .045), and the absence of NGT retention (OR 12.896; 95% CI 2.099, 79.219; *P* = .006) were independent risk factors predicting the development of a stricture.

**Conclusion:**

The combination of steroids with NGT retention is more effective than using oral steroids alone in preventing esophageal stricture after extensive ESD.

**Main Points**

- Oral steroids are effective in significantly reducing postoperative esophageal stricture incidence in patients with more than 3 quarters, but not complete, circumferential defects of the esophageal mucosa.
- However, the use of oral steroids alone demonstrates limited efficacy in preventing esophageal stricture after full circumferential resection.
- A favorable option for preventing esophageal stricture following total circumferential resection involves the combination of oral steroids with short-term nasogastric tube retention.

## Introduction

Endoscopic submucosal dissection (ESD) stands as the preferred treatment for superficial esophageal cancer to date. Studies have revealed that the en bloc resection rate and R0 resection rate of ESD for esophageal lesions all surpass 90%.^1^ Among the post-ESD complications of the esophagus, esophageal bleeding, esophageal perforation, and esophageal stricture are noteworthy, with esophageal stricture being the most prevalent.^2^ A previous research reported that the incidence of esophageal stricture after ESD for whole circumferential esophageal lesions can reach up to 100%.^3^ Furthermore, in patients with resection of esophageal mucosa exceeding three-quarters of the circumference, post-ESD esophageal stricture can induce symptoms such as dysphagia, nausea, vomiting, and other symptoms, significantly impacting patients’ quality of life.

The primary interventions for post-ESD esophageal stricture encompass balloon dilation, stent implantation, steroid-based therapy, etc. Balloon or bougie dilation, a traditional approach for esophageal stricture, involves a painful and multi-session process, posing economic burdens and compliance challenges for patients. Additionally, dilation may lead to complications such as esophageal bleeding and perforation.^4,5^ Stent displacement carries a high risk of esophageal stricture recurrence, and the safety and efficacy of new or specialized stents lack sufficient clinical validation.^6^ Mucosal autograft, an emerging technique, holds potential in preventing esophageal stricture, but its effectiveness requires further clarification, with 1 study reporting a 62.5% (5/8) esophageal stricture rate in patients undergoing full circumferential ESD.^7^

Steroids, due to their anti-inflammatory effects, offer multifaceted preventive mechanisms against esophageal stricture, including local inflammatory response inhibition, collagen synthesis inhibition, promotion of collagen decomposition, reduction of fibrous formation, and proline hydroxylase activity suppression, thus retarding tissue fibrosis. Steroid-based therapy stands as the primary prophylactic measure for postoperative stricture.^8^ Numerous studies attest to the efficacy and safety of steroids, their variants, or combinations in preventing esophageal stricture, with stricture rates ranging from 5.3% to 50% among patients with more than three-fourth circumferential defects.^9^ However, research indicates that steroids are ineffective in cases of whole circumferential defects, with one study reporting a stricture rate approaching 100%.^10^ Consequently, there is a need for novel measures in stricture prophylaxis after extensive ESD.

The nasogastric tube (NGT), a soft and opaque drainage tube for nutrient delivery or intragastric pressure relief through the nose into the stomach, distinguishes itself by lower susceptibility to displacement compared to other methods.^11^ To date, there is no reported effect of NGT retention in preventing esophageal stricture. While NGT retention may entail patient discomfort or associated risks, clinical observations suggest that short-term NGT retention may indeed positively impact reducing esophageal stricture post-ESD. Thus, this study aims to compare the efficacy of oral prednisolone alone with oral prednisolone plus nasogastric tube in preventing esophageal stricture following endoscopic submucosal dissection in superficial esophageal cancer.

## Materials and Methods

### Study Population

This retrospective study included patients diagnosed with superficial esophageal cancer who underwent extensive ESD at a single center (Affiliated Jinhua Hospital of Zhejiang University School of Medicine) from January 2017 to January 2022. Preoperative magnification endoscopy with narrow-band imaging and endoscopic ultrasonography was conducted to confirm lesion architecture and exclude esophageal cancer with submucosal invasion. Patients with more than a three-fourth circumferential defect, including full circumferential defects, were eligible for inclusion. Exclusion criteria comprised (1) patients undergoing additional therapies such as radiotherapy, chemotherapy, and surgery; (2) patients with esophageal stricture before ESD; (3) patients with heart, liver, kidney, or other essential organ dysfunctions incompatible with treatment; (4) contraindications for steroid use and nasogastric tube placement. The study adhered to the Declaration of Helsinki and was approved by the Zhejiang University Jinhua Hospital Medical Science Research Ethics (approval number: 56, date: 2022). All patients provided written informed consent.

Based on prophylactic measures, patients were categorized into 2 groups: the steroid group (patients received oral prednisolone for 8 weeks) and the steroid + NGT group (patients received oral prednisolone for 8 weeks combined with nasogastric tube retention for 4 weeks). Patients with more than three-fourth circumferential extension defect or intolerance of nasogastric tube retention were recommended to the steroid group. Patients with nearly total circumferential defects but without contraindications to nasogastric tube retention were more suitable for the steroid + NGT group.

### Endoscopic Submucosal Dissection Procedure

**Figure 1.**
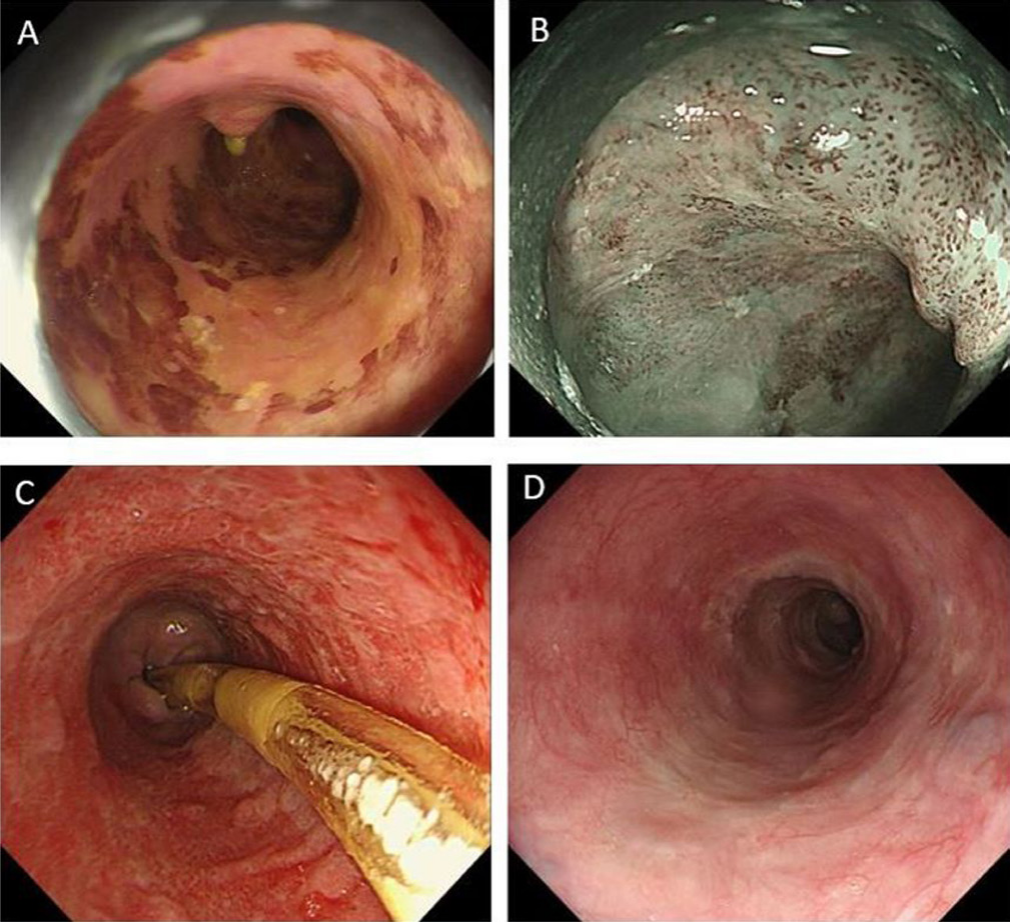
Endoscopic views of the esophagus of a case in the steroid + NGT group. (A) Chromoendoscopy with iodine staining shows a discolored area near whole circumference in the middle esophagus. (B) Magnifying endoscopy combined with a narrow-band imaging system suggest a nonsubmucosal infiltration cancer. (C) Nasogastric tube retention after ESD for 4 weeks. (D) Oral prednisolone for 8 weeks and nasogastric tube retention for 4 weeks. Endoscopic review at 12th week reveals no esophageal stricture.

All ESD procedures were performed under general anesthesia and tracheal intubation by experienced physicians. Lesions were initially observed under white light and narrow-band imaging to assess invasion depth. Subsequently, the lesion was stained with iodine and marked 1 mm outside the lesion margin. Submucosal injection of glycerin fructose and methylene blue mixed solution was administered at multiple points to fully elevate the lesion. Mucosal incision and submucosal dissection were then carried out, followed by electrocoagulation to manage bleeding. Postoperatively, a nasogastric tube was inserted under endoscopy with a drainage bag connection (approximately 4.7 mm in diameter, 75 cm in scale length), with about 55 cm of the tube inserted. The circumferential extent of the mucosal defect after ESD was classified into 2 groups: more than three-quarters but not complete (≥3/4) and complete circumferential defect.^1^ Figure 1 illustrated the endoscopic views of the esophagus in a case from the steroid + NGT group.

### Postprocedural Management

Patients received oral prednisolone starting on the third day after ESD at a dose of 30 mg/day, gradually tapering off (30, 30, 25, 25, 20, 15, 10, and 5 mg for 7 days each), and discontinued after 8 weeks.^12^ On the second day after ESD, the drainage bag was removed, and the nasogastric tube was sealed. Patients could then be discharged with the nasogastric tube in place. In the steroid + NGT group, the nasogastric tube was removed by physicians during follow-up at 4 weeks.

### Follow-up Protocol

Patients underwent follow-up at the outpatient department at 2, 4, 8, and 12 weeks post ESD. Two trained physicians assessed dysphagia or other symptoms, and a gastroscopy was conducted at 4 weeks. Esophageal stricture was defined as a narrow esophageal cavity impeding passage with a standard endoscope (approximately 9 mm in diameter) after ESD, causing dysphagia symptoms.^13^ Common postoperative complications encompassed fever, hemorrhage, and perforation. Steroid-related complications comprised peptic ulceration, infections, diabetes, etc. Nasogastric tube retention-related adverse events included esophageal or pharyngeal injury, unplanned tube removal, and discomfort. The depth of histological invasion of esophageal cancer was categorized into 4 groups following WHO criteria.^14^

### Statistical Analysis

Continuous variables were presented as mean ± SD and compared using Student’s *t*-test. Categorical variables were presented as percentages and analyzed using Pearson chi-square test, Fisher’s exact test, or the continuity correction Chi-square test. Logistic regression analysis determined independent risk factors for stricture. Variables with *P*<.1 in univariate analysis using chi-square test or Student’s *t*-test were included in the multivariate logistic regression analysis. *P* values < .05 were considered statistically significant. Statistical analyses were conducted using the Statistical Package for Social Sciences (SPSS) software (version 25, IBM Corp., Armonk, NY, USA).

## Results

### General Information of the Patients

The study included 79 patients, with 30 in the steroid group and 49 in the steroid + NGT group. There was no significant difference in the basic clinical characteristics of the patients, including gender, age, lesion location in the esophagus, and mucosal defect length, between the steroid and steroid + NGT groups (*P*>.05) (Table 1). Fever was the most common post-ESD complication, with no serious complications like hemorrhage or perforation observed in either group (Table 2). Additionally, there were no complications related to prednisolone occurred during the follow-up. In the NGT group, 4 (8.16%) patients experienced discomfort and removed the tube without planned intervention.

### Comparison of the Steroid and Steroid + NGT Groups Based on the Presence of a Circumferential Defect

The stricture incidence in the steroid group (9/30, 30.0%) was significantly higher than in the steroid + NGT group (3/49, 6.1%) (*P* = .004) (Table 2). For patients with a whole circumference defect, the stricture rate was significantly higher in the steroid group (100%, 5/5) compared to the NGT group (16.7%, 1/6) (*P* = .015). However, there was no statistical significance in the stricture rate between the steroid group and NGT group for patients with three-quarter circumference defects (Table 3).

### Risk Factors for Esophageal Stricture after Endoscopic Submucosal Dissection

Preliminary analysis indicated potential associations between esophageal stricture and independent predictive variables, including circumferential mucosal defect, invasion depth, and nasogastric tube retention (Table 4). Multivariate logistic regression analysis identified circumferential defect of the esophageal mucosa as an independent risk factor for stricture (OR 12.501; 95% CI 1.907, 81.047; *P* = .008), deeper than M2 invasion depth as another risk factor (OR 5.635; 95% CI 1.039, 30.559; *P* = .045), and the absence of nasogastric tube retention as a factor increasing the risk of stricture (OR 12.896; 95% CI 2.099, 79.219; *P* = .006).

## Discussion

This study represents the first known research to compare the effectiveness of oral prednisolone alone versus oral prednisolone combined with nasogastric tube in preventing esophageal stricture among patients undergoing more than three-fourth circumferential ESD. Our findings demonstrated that prophylaxis with oral prednisolone plus nasogastric tube retention exhibits superior efficacy in preventing esophageal stricture compared to oral prednisolone alone in patients undergoing complete resection.

Endoscopic mucosal dissection is the preferred treatment for superficial esophageal cancer; however, esophageal stricture is prone to occur in patients with near circumferential ESD. The incidence of post-ESD stricture was notably high, reaching 94.1% for patients with more than three-fourth circumferential range ESD, and nearly 100% after whole circumferential ESD without prophylactic measures.^12,14^ Steroids have demonstrated efficacy in reducing inflammatory processes, collagen synthesis, and fibroblast proliferation, promoting fibroblast degeneration, and thereby inhibiting stricture formation.^15^ Steroid administration remains the most common intervention for esophageal stricture after ESD. Prior studies indicated stricture rates of 5.3%-23.1% in patients with more than three-fourth circumferential extension defects after steroid use.^12,16^ However, even in the most optimistic research, a 50% stricture rate after full circumferential ESD persists despite oral steroids.^17^ In our study, the stricture incidence of 30.0% (9/30) with oral steroids was slightly higher than previous studies, possibly due to the inclusion of patients with full circumferential mucosal defects. Notably, in the steroid group, all 5 patients undergoing total circumferential resection developed stricture, resulting in a 100% stricture rate (5/5). In contrast, the stricture rate for nontotal resection was only 16.0% (4/25). Therefore, steroid administration may be a beneficial approach for the patients with nontotal resection after ESD at risk of stricture, but not for those after whole circumferential ESD. The differences in epithelial regeneration between total circumferential defect and nontotal circumferential defect might contribute to this variation. For total circumferential resection, the lack of a continuous epithelial layer and heightened inflammation could be contributing factors.^10^

In patients undergoing large circumferential ESD, the steroid plus NGT group exhibited a significantly lower stricture rate (3/49: 6.1%) compared to those receiving only oral steroids (9/30:30%) (*P* = .004). Notably, NGT retention played a positive role in patients with whole circumferential esophageal resection, yielding a stricture rate of 16.7% in the combined group compared to 100% in the steroid group (*P* = .015). These findings suggest that the combination of oral steroids and nasogastric tube retention may be an effective option for preventing esophageal stricture after whole circumferential ESD. The mechanism of esophageal stricture post ESD is intricate, potentially involving damage to the muscularis layer leading to submucosal fibrosis of the esophagus.^18^ It is hypothesized that the lack of support from the esophageal muscular layer may predispose to stricture. The nasogastric tube is proposed to function by supporting and stabilizing the esophageal wound, preventing mucosal surfaces from adhering and fusing, thereby alleviating stenosis formation. However, these hypotheses necessitate further confirmation through additional studies.

Previous studies have indicated potential risks associated with nasogastric tube retention, including pneumonia, reinsertion complications, and patient discomfort.^19^ Unfortunately, 4 patients (8.16%) with nasogastric tube retention experienced significant discomfort in this study. To address this issue, comprehensive patient education and care, including secure tube fixation, psychological counseling, and adopting a semirecumbent position after meals, were deemed essential. Additionally, the use of mucosal protectants and antireflux medications such as “almagate suspension” may help alleviate discomfort. Simultaneously, combined oral steroids could mitigate mucosal edema and inflammation. No other related complications were identified during follow-up, indicating that short-term nasogastric tube retention was relatively safe. Nevertheless, further studies are warranted to confirm the effects and determine the most appropriate duration of this intervention.

According to previous reports, histological invasion depth and lesion range may serve as independent predictors of post-ESD stricture.^14,20^ Our study supports these findings, demonstrating through multivariate analysis that both full circumferential extension (OR 12.501; 95% CI 1.907, 81.047; *P *= .008) and invasion depth beyond M2 (OR 5.635; 95% CI 1.039, 30.559; *P *= .045) independently contribute as risk factors. The diminished thickness or damage to the muscularis layer, leading to the loss of muscle fibers, could make it challenging to maintain the anatomical structure of the esophagus post ESD.^21^ Additionally, in cases of full circumferential resection, epithelial regeneration occurs only from the proximal and distal sides of lesions, whereas in noncircumferential resection, regeneration occurs from both the proximal and distal sides and the longitudinal residual normal mucosa.^22^ These factors may contribute to the increased likelihood of esophageal stricture in cases with large circumferential defects and invasion depth reaching M3. The formation of esophageal stricture is a complex process involving chemical, immune, and other factors, warranting further investigation.

The study had some limitations. First, potential risks associated with steroids and nasogastric tube retention were not fully addressed. Secondly, being a retrospective, single-center study, there is inherent selection bias, and many clinical data records are incomplete or missing. Additionally, this small-sample study may have systematic errors, and its conclusions may not be extrapolated to the general population. Further multicenter and large-sample randomized controlled trials are necessary to validate the conclusions.

In conclusion, the combination of nasogastric tube retention plus oral steroids demonstrated superior efficacy compared to oral steroids alone (stricture rate 6.1% vs. 30.0%) in preventing esophageal stricture post ESD, particularly in patients with whole circumferential defects. Circular extents of resection, invasion depth, and prophylaxis measures were identified as independent risk factors for the development of esophageal stricture.

## Figures and Tables

**Table 1.** Patient Information and Details between Steroid Group and Steroid + NGT Group

Characteristics		Steroid group, (n = 30) n (%)	Steroid + NGT group, (n = 49) n (%)	*P*
Gender	Male	19 (63.3%)	37 (75.5%)	.248
	Female	11 (36.7%)	12 (24.5%)	
Age, years		69.40 ± 6.64	68.55 ± 6.98	.594
Lesion location of esophagus	Upper	5 (16.7%)	6 (12.2%)	.692
	Middle	18 (60.0%)	34 (69.4%)	
	Lower	7 (23.3%)	9 (18.4%)	
Mucosal defect length, cm		4.13 ± 1.36	4.31 ± 1.69	.636
Circumference of mucosal defect	≥3/4	25 (83.3%)	43 (87.8%)	.582
	1	5 (16.7%)	6 (12.2%)	

NGT, nasogastric tube.

**Table 2.** Patient Information after Endoscopic Submucosal Dissection

Characteristics	Steroid Group, (n = 30) n (%)	Steroid + NGT Group, (n = 49) n (%)	P
Invasion depth			
				M1, M2	19 (63.3%)	26 (53.1%)	.371
M3, SM1	11 (36.7%)	23 (46.9%)	
Stricture			
				Yes	9 (30.0%)	3 (6.1%)	.004
No	21 (70.0%)	46 (93.9%)	
Postoperative complication			
Hemorrhage	0(0)	0(0)	
Fever	7 (23.3%)	9 (18.4%)	
Perforation	0 (0)	0 (0)	
Steroid-related	0 (0)	0 (0)	
NGT-related	0 (0)	4 (8.16%)	

Steroid-related complications included peptic ulceration, infections, diabetes, and so on. NGT-related adverse events included esophageal or pharyngeal injury, unplanned tube removal and discomfort.

M1, the cancer situated in the epithelial lining; M2, the cancer infiltration into the lamina propria; M3, the cancer invasion into the muscularis mucosa; NGT, nasogastric tube; SM1, the cancer infiltration into the submucosa to a depth of less than 1/3.

**Table 3.** Comparison between Steroid Group and Steroid + NGT Group According to Circumferential Defect

	Three-Quarter Circumference		Whole Circumference	
	Stricture, n (%)	No stricture, n (%)	Stricture, n (%)	No stricture, n (%)
Steroid group	4 (16.0%)	21 (84.0%)	5 (100%)	0 (0%)
Steroid + NGT group	2 (4.7%)	41 (95.3%)	1 (16.7%)	5 (83.3%)
*P*	.112		.015	

NGT, nasogastric tube.

**Table 4:** Logistic Regression Analysis of Risk Factors for Esophageal Stricture

Variables	Stricture n (%)	Univariate Analysis		Multivariate Analysis		
		t/χ^2^	*P*	OR	95%CI	*P*
Gender						
							Male	9 (75.0%)	.116	.733	–	–	–
Female	3 (25.0%)					
Age, years	70.50 ± 4.43	−1.317	.192	–	–	–
Lesion location in the esophagus						
							Upper	0 (0.0%)	2.719	.257	–	–	–
							Middle	10 (83.3%)					
Lower	2 (16.7%)					
Mucosal defect length, cm	4.93 ± 1.21	−1646	.104	–	–	–
Circumference of mucosal defect						
							1	6 (50.0%)	15.365	<.001	12.501	1.907, 81.047	.008
≥3/4	6 (50.0%)					
Invasion depth						
							M3, SM1	9 (75.0%)	5.896	.015	5.635	1.039, 30.559	.045
M1, M2	3 (25.0%)					
Nasogastric tube retention						
							No	9 (75.0%)	8.235	.004	12.896	2.099, 79.219	.006
Yes	3 (25.0%)					

M1, the cancer situated in the epithelial lining; M2, the cancer infiltration into the lamina propria; M3, the cancer invasion into the muscularis mucosa; OR, odd ratio; SM1, the cancer infiltration into the submucosa to a depth of less than one-third.
